# The Association of Childhood Pneumonia with Household Air Pollution in Nepal: Evidence from Nepal Demographic Health Surveys

**DOI:** 10.1007/s10995-020-02882-x

**Published:** 2020-01-24

**Authors:** Shyam Sundar Budhathoki, Bhim Singh Tinkari, Amit Bhandari, Meghnath Dhimal, Hong Zhou, Anup Ghimire, Omkar Basnet, Johan Wrammert, Ashish KC

**Affiliations:** 1 School of Public Health and Community Medicine, B.P. Koirala Institute of Health Sciences, Dharan, Nepal; 2 Golden Community, Lalitpur, Nepal; 3 Ministry of Health and Population, Government of Nepal, Kathmandu, Nepal; 4 Society of Public Health Physicians Nepal, Kathmandu, Nepal; 5 Nepal Health Research Council, Kathmandu, Nepal; 6 Department of Maternal and Child Health, School of Public Health, Peking University, Beijing, China; 7 Department of Women’s and Children’s Health, Uppsala University, Uppsala, Sweden

**Keywords:** Childhood pneumonia, Nepal, Solid fuels, Polluting fuels, Indoor air pollution

## Abstract

**Introduction:**

Childhood pneumonia is a major cause of mortality worldwide while household air pollution (HAP) is a major contributor to childhood pneumonia in low and middle-income countries. This paper presents the prevalence trend of childhood pneumonia in Nepal and assesses its association with household air pollution.

**Methods:**

The study analysed data from the 2006, 2011 and 2016 Nepal Demographic Health Surveys (NDHS). It calculated the prevalence of childhood pneumonia and the factors that cause household air pollution. The association of childhood pneumonia and HAP was assessed using univariate and multi-variate analysis. The population attributable fraction (PAF) of indoor pollution for causing pneumonia was calculated using 2016 NDHS data to assess the burden of pneumonia attributable to HAP factors.

**Results:**

The prevalence of childhood pneumonia decreased in Nepal between 2006 and 2016 and was higher among households using polluting cooking fuels. There was a higher risk of childhood pneumonia among children who lived in households with no separate kitchens in 2011 [Adjusted risk ratio (ARR) 1.40, 95% CI 1.01–1.97] and in 2016 (ARR 1.93, 95% CI 1.14–3.28). In 2016, the risk of children contracting pneumonia in households using polluting fuels was double (ARR 1.98, 95% CI 1.01–3.92) that of children from households using clean fuels. Based on the 2016 data, the PAF for pneumonia was calculated as 30.9% for not having a separate kitchen room and 39.8% for using polluting cooking fuel.

**Discussion for Practice:**

Although the occurrence of childhood pneumonia in Nepal has decreased, the level of its association with HAP remained high.

## Significance

Childhood pneumonia is an important cause of under-five mortality while household air pollution (HAP) is a major risk factor for under-five mortality and childhood pneumonia. The use of polluting fuel is an important risk factor for childhood pneumonia in Nepal. This paper adds information on the trend of childhood pneumonia and its association with HAP in addition to the use of polluting fuels. It also presents the population attributable fraction (PAF) of HAP factors to childhood pneumonia from the 2016 NDHS.

## Introduction

Globally, 120 million cases of childhood pneumonia were reported in 2010 (Walker et al. 2013). Fourteen million of these cases progressed to severe episodes and 47.4 million cases were reported from South Asia (Walker et al. 2013). In 2011, 1.3 million under-5-year-olds died from pneumonia, with a third of these deaths occurring in South Asia (Rudan et al. 2013). A high proportion (81%) of these deaths occurred in children under 2 years of age (Walker et al. 2013). In 2010, there were 0.8 million episodes of childhood pneumonia in Nepal that occurred in under-5-year-olds with 95,000 of these episodes progressing to severe pneumonia and 5500 of these children die (Rudan et al. 2013). The incidence of childhood pneumonia in Nepal has reduced considerably from 244 per 1000 under-5-year-olds in 2014 to 147 per 1000 under-5-year-olds in 2016 (Ministry of Health & Population 2017).

Several risk factors such as malnutrition, low birth weight, non-exclusive breastfeeding, overcrowding at home and the use of polluting cooking fuel are the major contributors to childhood pneumonia in low and middle income countries (LMICs) (Naz et al. 2018a, b; Quansah et al. 2017). The use of polluting cooking fuel is mainly responsible for HAP in LMICs (Quansah et al. 2017).

Solid fuels are the main source of cooking fuel for 41% of households worldwide (Bonjour et al. 2013). Solid fuel for cooking, heating and lighting is derived from coal or plant materials (biomass) by about a third of the world’s population (Naz et al. 2018b). Three billion people worldwide are exposed to toxic amounts of HAP on a daily basis and one-third of this population resides in South Asia (Bonjour et al. 2013). HAP is associated with upper and lower respiratory tract infections (Gordon et al. 2014). It is estimated that globally 455,000 pneumonia deaths annually are due to HAP, which leads to the loss of 39,100,000 disability-adjusted life-years (DALYs) (GBD 2016 Risk Factors Collaborators, 2017). The PAF of pneumonia cases due to HAP has been calculated at 52% (Gakidou et al. 2017).

Nepal in its most recent population census reported a population of 27.5 million, with 83% living in rural areas (Central Bureau of Statistics, Nepal 2014). Solid fuels were used for household cooking in 64% of households (Central Bureau of Statistics, Nepal 2014). Common solid fuels include dried animal dung, wood, coal and crop residues (Gordon et al. 2014). In Nepal, it is mostly women and children who are exposed to HAP as women do most household work including cooking (Pant 2012).

One study reported the use of kerosene (OR 1.87, 95% CI 1.24–2.83) and solid fuels (1.93, 1.24–2.98) for cooking as risk factors for childhood pneumonia in Nepal (Bates et al. 2013). Another study in Nepal conducted in the hill district of Dhading, attributed 50% of childhood pneumonia cases to cooking with solid fuels (Dhimal et al. 2010). Despite an availability of large datasets from the Nepal Demographic Health Survey being available every 5 years, reports on the trends of prevalence of childhood pneumonia, HAP factors and their association are not found in the literature. Knowing how these trends have changed over the years could support relevant stakeholders and provide potential points to consider while formulating future policies to alleviate HAP and childhood pneumonia in Nepal. The current study presents the prevalence trends of childhood pneumonia and HAP factors in 2006, 2011 and 2016 and the association of childhood pneumonia with HAP in Nepal.

## Methods

This study carried out analysis of datasets from the 2006, 2011 and 2016 Nepal Demographic Health Surveys (Ministry of Health and Population et al. 2007, 2012; Ministry of Health et al. 2017). The NDHS is a nationwide cross-sectional survey that is conducted every 5 years. These questionnaire-based surveys are administered to nationally representative samples that are selected using multistage cluster random sampling. The datasets were obtained from the DHS program website.

### Study Setting

Nepal is a low-income country with 7% of its population in mountain districts, 43% in hill districts and 50% in the Terai plains in 2011. 85% live in rural areas. In 2016, the crude birth rate was 22.4/1000 people, the death rate 6.3/1000 people, the maternal mortality ratio 239/100,000 live births, the neonatal mortality rate 21/1000 live births, the infant mortality 32/1000 live births and the child mortality rate 39/1000 live births (Ministry of Health et al. 2017).

### Data Collection

The 2006, 2011 and 2016 NDHS covered all Nepal’s districts, which were stratified into urban (municipality) and rural (village development committee) areas. In the first stage, particular wards or sub-wards (enumeration areas) were selected using probability proportional to size. Households were then selected from each enumeration area, or from a segment of them for participation in the survey. The details of the sample selection process have been elsewhere mentioned (Ministry of Health and Population et al. 2007, 2012; Ministry of Health et al. 2017). There was also the non-proportional allocation of samples of different sampling domains, and oversampling in urban areas; which required weighted analysis to ensure representation at national and domain levels. The data used in this study were collected by trained enumerators administering the DHS women’s questionnaire.

For current purposes, information from the women’s questionnaires, recoded in two datasets (viz. children under-5 recode [KR] and household recode [HR]), were merged for each survey year and used. This provided information on 5457 under-5-year-old children from the 2006 NDHS, 5054 such children from the 2011 NDHS and 4861 such children from the 2016 NDHS.

### Variables

The main outcome variable in this study was childhood pneumonia, which was defined by the NDHS as the presence of symptoms of acute respiratory infection (ARI) among under-5-year-old children during the 2 weeks preceding the survey.

The exposure variables were socio-demographic characteristics, nutritional status and HAP factors. The socio-demographic factors were the age of the mother, mother’s education, mother’s occupation, mother’s tobacco smoking status, sex of the child, area of residence, the presence of a toilet at home and wealth quintile. The nutritional status of the child was classified as stunting, wasting, underweight and size at birth. The HAP-related information was type of household flooring, the presence of tobacco smoking inside the house, having a separate kitchen room and type of cooking fuel.

For analysis, the study re-categorized the wealth quintile variables into either poor (= poorest and poorer quintiles) or non-poor (= middle, second wealthiest and wealthiest quintiles). Residence was categorized as either rural or urban with urban residence as the reference variable in the univariate and multivariate analysis. The ecological region was either Terai, hill or mountain with Terai as the reference category. Mother’s age was categorized into 15–24 years, 25–34 years and 35 years and above while mother’s education was categorized into none or at least primary education. The other variables were mother being in paid employment or not, households having a toilet facility or not, the gender and nutritional status of the child (under-nourished, wasted, stunted), and small size at birth, and the HAP variables of house flooring type, tobacco smoking inside the house (second hand smoke exposure), separate kitchen space, and main type of cooking fuel. Note that data on smoking tobacco inside the house were not available in the 2006 NDHS and clean fuels were defined as electricity, LPG (liquefied petroleum gas), biogas and natural gas, and polluting fuels as kerosene, wood, straw, shrubs, grass, animal dung, coal and charcoal.

### Data Analysis

The trends of the prevalence of childhood pneumonia and the HAP factors were plotted on a time series chart from 2006, 2011 and 2016. The association between socioeconomic variables and HAP indicators with childhood pneumonia was determined using Pearson’s *χ*^2^ test. Simple logistic regression was applied to calculate the crude risk ratio for the variables. All HAP variables, and the socio-demographic and nutrition variables associated with childhood pneumonia at p ≤ 0.1 were considered for multivariable regression analysis.

The PAF of the HAP for causing pneumonia was calculated using the most recent available data—the NDHS 2016 data. The burden of childhood pneumonia attributable to HAP was calculated as the PAF, assuming independently distributed exposures and independent hazards (Ezzati et al. 2003). The following formula was used to calculate the PAF:

$$PAF = \frac{{P_{exp } *\left( {RR - 1} \right)}}{{P_{exp } * \left( {RR - 1} \right) + 1}}$$

In the formula: P_exp_ = proportion of HAP exposure and RR = relative risk of pneumonia among exposed to unexposed.

## Results

### Trend of Childhood Pneumonia

**Fig. 1:**
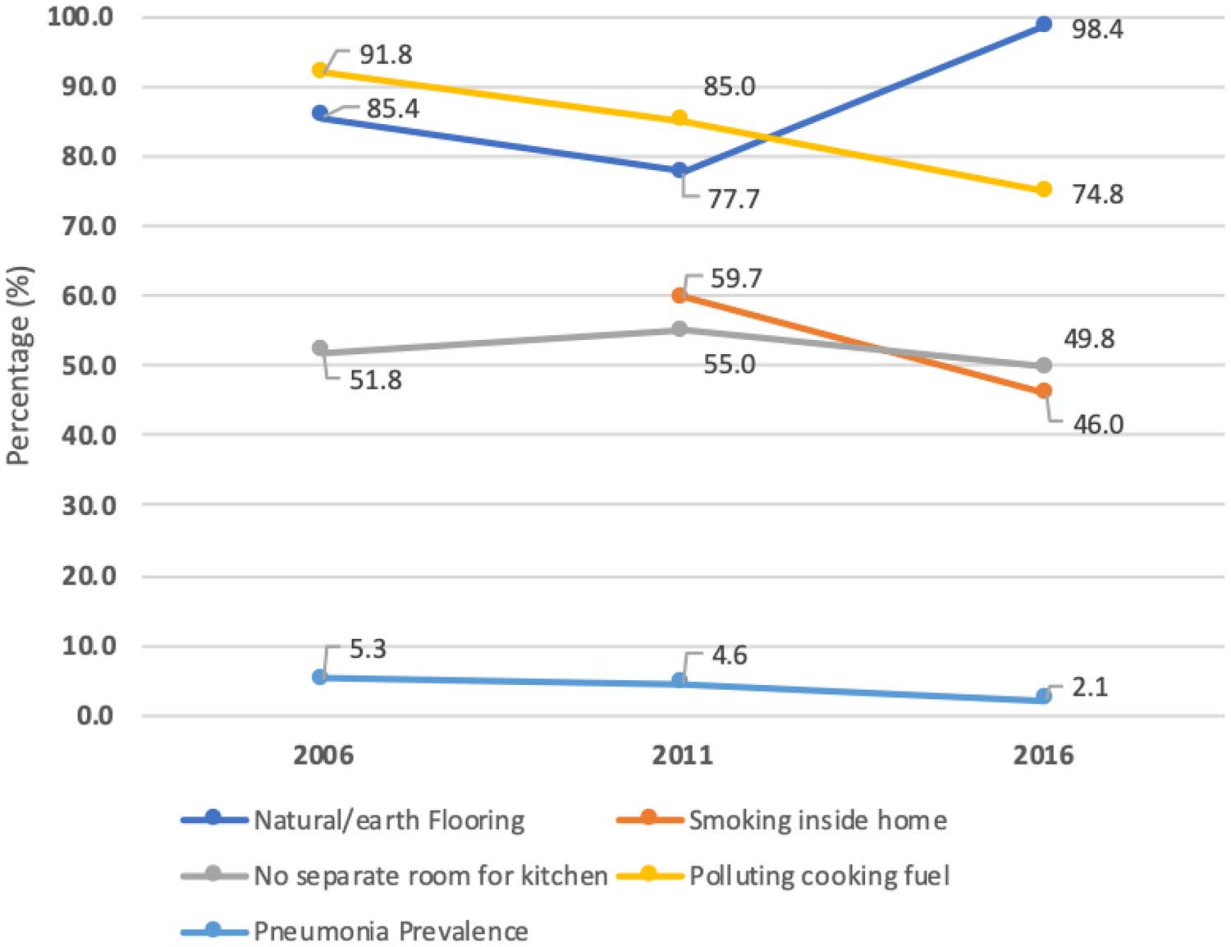
Prevalence trend of HAP factors and childhood pneumonia in 2006, 2011 and 2016 in Nepal (NDHS data)

The prevalence of childhood pneumonia declined from 5.3% in 2006 to 2.1% in 2016. Among the HAP factors, the prevalence of smoking inside homes had decreased from 59.7% in 2011 to 46.0% in 2016); the proportion of households with no separate kitchen room had decreased from 51.8% in 2006 to 49.8% in 2016; the use of polluting fuels for cooking had decreased from 91.8% of households in 2006 to 74.8% in 2016; and the proportion of children living in houses with natural floors (earth/sand or dung) increased from 85.4% in 2006 to 98.4% in 2016 (Fig. 1).

**Fig. 2:**
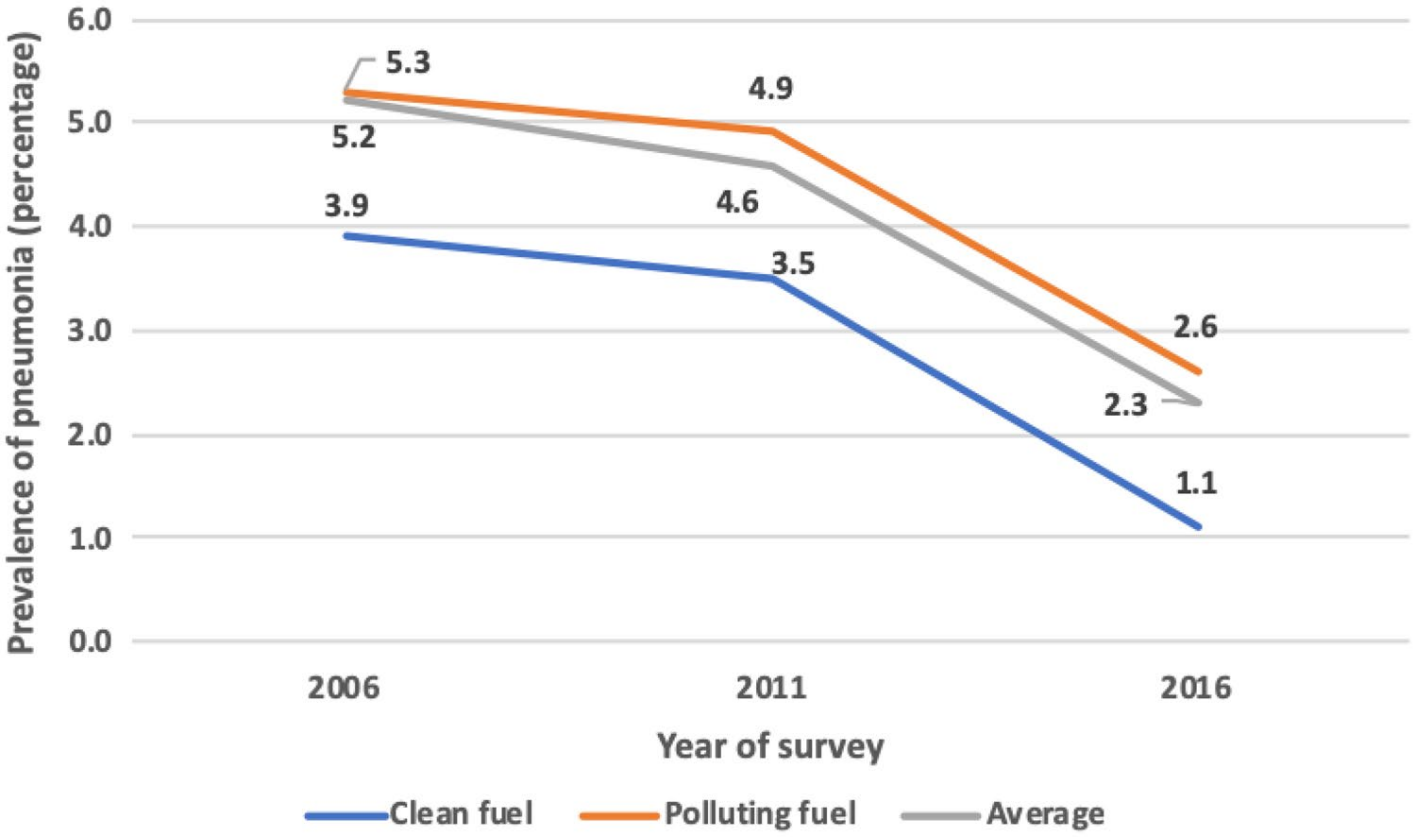
Trend of childhood pneumonia according to the type of cooking fuel used in NDHS households in 2006, 2011 and 2016

The prevalence of childhood pneumonia among households using polluting fuels was 1–2% higher compared to households using clean fuels in all three surveys (2006, 2011 and 2016) (Fig. 2).

### Univariate Analysis

The results of the univariate analysis across the three NDHS were as follows:

- In 2006, a higher proportion of pneumonia was reported in children whose mothers had formal education (p < 0.01), whose mothers were employed (p < 0.01), were living in the mountain and hill regions (p = 0.01), and who had wasting (p = 0.03).
- In 2011, higher proportions of pneumonia were reported in children whose mothers were employed (p = 0.09) and who were small size at birth (p = 0.07), however these were not significant associations.
- In 2016, a higher proportion of pneumonia was reported in children from the mountain and hill regions (p < 0.01), whose households had a toilet (p < 0.01), who were from poor families (p < 0.01) and who were small sized at birth (Table 1).

**Table 1:** Socio-demographic factors associated with childhood pneumonia (NDHS data, 2006–2016)

Characteristics	Childhood pneumonia					
	2006	p-value	2011	p-value	2016	p-value
Age of mother						
15–24	134/2160 (6.2%)	0.24	113/2071 (5.5%)	0.06	49/1973 (2.5%)	0.12
25–34	111/2415 (4.6%)		102/2462 (4.1%)		44/2510 (1.8%)	
≥ 35	32/677 (4.7%)		23/607 (3.8%)		12/404 (3%)	
Mother’s formal education						
No	145/3129 (4.6%)	<0.01	106/2409 (4.4%)	0.46	32/1664 (1.9%)	0.43
Yes	132/2123 (6.2%)		132/2730 (4.8%)		73/3223 (2.3%)	
Mother’s occupation						
Not employed	30/1041 (2.9%)	<0.01	57/1483 (3.8%)	0.09	33/1995 (1.7%)	0.06
Employed	246/4210 (5.8%)		181/3656 (5%)		71/2891 (2.5%)	
Mother’s smoking status						
Smoker	63/1013 (6.2%)	0.13	33/653 (5.1%)	0.58	9/278 (3.2%)	0.2
Non-smoker	214/4239 (5%)		205/4486 (4.6%)		96/4609 (2.1%)	
Residence						
Urban	33/652 (5.1%)	0.8	24/484 (5%)	0.73	47/2649 (1.8%)	0.05
Rural	244/4600 (5.3%)		215/4687 (4.6%)		58/2239 (2.6%)	
Ecological zone						
Mountain	28/443 (6.3%)	0.01	13/401 (3.2%)	0.19	9/342 (2.6%)	<0.01
Hill	136/2171 (6.2%)		105/2032 (5.2%)		58/1857 (3.1%)	
Terai	113/2638 (4.3%)		120/2707 (4.4%)		38/1687 (1.4%)	
Toilet at home						
Present	112/1976 (5.7%)	0.21	106/2349 (4.5%)	0.68	91/3484 (2.6%)	<0.01
Absent	144/2965 (4.9%)		117/2455 (4.8%)		11/1030 (1.1%)	
Wealth quintile						
Poor	128/2445 (5.2%)	0.24	115/2443 (4.7%)	0.85	60/2069 (2.9%)	<0.01
Non-poor	149/2807 (5.3%)		124/2697 (4.6%)		45/2818 (1.6%)	
Sex of child						
Male	151/2681 (5.6%)	0.24	122/2649 (4.6%)	0.93	60/2564 (2.3%)	0.33
Female	126/2571 (4.9%)		116/2490 (4.7%)		45/2323 (1.9%)	
Child stunted						
No	142/2551 (5.6%)	0.54	81/1486 (5.5%)	0.89	31/1518 (2%)	0.38
Yes	128/2474 (5.2%)		51/958 (5.3%)		22/845 (2.6%)	
Child underweight						
No	165/3091 (5.3%)	0.89	96/1764 (5.4%)	0.78	41/1729 (2.4%)	0.33
Yes	105/1934 (5.4%)		35/679 (5.2%)		11/641 (1.7%)	
Child wasted						
No	223/4387 (5.1%)	0.03	117/2183 (5.4%)	0.79	48/2129 (2.3%)	0.93
Yes	46/637 (7.2%)		15/261 (5.7%)		5/231 (2.2%)	
Small size at birth^a^						
No			191/4331 (4.4%)	0.07	79/4062 (1.9%)	0.04
Yes			47/803 (5.9%)		25/818 (3.1%)	

^a^Not available in 2006 NDHS

In 2006, the HAP factors were not associated with childhood pneumonia while in 2011, higher proportions of pneumonia were reported among children exposed to tobacco smoke in their homes (p = 0.03) and whose homes had no separate kitchen room (p = 0.03). In 2016, higher proportions of pneumonia were reported in children whose homes had no separate kitchen (p = 0.04) and where polluting fuels were used for cooking (p < 0.01) (Table 2).

**Table 2:** Household air pollution-related environmental factors and their association with pneumonia (NDHS data, 2006–2016)

Characteristics	Categories	Childhood pneumonia					
		2006	p-value	2011	p-value	2016	p-value
Flooring	Natural (earth, sand, dung)	218/4179 (5.2%)	0.73	179/3733 (4.8%)	0.35	82/3096 (2.6%)	0.64
	Cement/carpet	35/714 (4.9%)		44/1069 (4.1%)		0/52 (0%)	
Cigarette smoking inside the house^a^	No	–	– –	80/2073 (3.9%)	0.03	58/2641 (2.2%)	0.72
	Yes	–		158/3067 (5.2%)		46/2245 (2%)	
Separate room for kitchen	No	121/2122 (5.7%)	0.75	110/1975 (5.6%)	0.03	41/1587 (2.6%)	0.04
	Yes	108/1973 (5.5%)		65/1618 (4%)		25/1603 (1.6%)	
Main cooking fuel	Clean	16/407 (3.9%)	0.23	25/721 (3.5%)	0.1	13/1140 (1.1%)	< 0.01
	Polluting	240/4533 (5.3%)		198/4081 (4.9%)		89/3375 (2.6%)	

^a^Not available for 2006

### Multivariate Analysis

The multivariate analysis found for 2006 that children living in the hill region had a 38% higher risk of having childhood pneumonia (ARR 1.38, confidence interval [CI] 95% 1.06–1.81) than children in the Terai (Table 1). The risk of having pneumonia were even higher in 2016 with children in the hills having 109% higher risk (ARR-2.09, CI 95% 1.08–4.03) than children in the Terai. In 2011, there was 40% risk of childhood pneumonia among children who lived in households with no separate kitchens (ARR-1.40, 95% CI 1.01–1.97). In 2016, children in households without a separate kitchen had 93% higher risk of having pneumonia (ARR-1.93, CI 95%, 1.14–3.28) while children in households using polluting fuel had a 98% higher risk (ARR-1.98, CI 95% 1.01–3.92) (Table 3).

**Table 3:** Level of association of different risk factors with childhood pneumonia in 2006, 2011 and 2016 (NDHS data)

Characteristics	Category	2006	2011	2016
		ARR (95% CI)	ARR (95% CI)	ARR (95% CI)
Mother’s age	25–34 years		Reference	
	15–24 years		1.28 (0.91–1.79)	–
	35 years & above		0.82 (0.48–1.40)	–
Mother formally educated	Yes	Reference		
	No	0.68 (0.53–0.88)	–	–
Mother in paid employment	No	Reference	Reference	Reference
	Yes	1.95 (1.30–2.91)	1.47 (0.94–2.28)	1.23 (0.65–2.29)
Ecological region	Terai	Reference		Reference
	Mountains	1.39 (0.89–2.17)	–	1.60 (0.62–4.10)
	Hills	1.38 (1.06–1.81)	–	2.09 (1.08–4.03)
Toilet facility at home	Yes	–	–	Reference
	No	–	–	0.53 (0.23–1.25)
Wasting status of child	Normal	Reference		
	Wasted	1.61(1.16 –2.26)	–	–
Small size at birth	No	Not available	Reference	Reference
	Yes	Not available	1.21 (0.81–1.80)	1.38 (0.77–2.48)
Indoor smoking of tobacco	No	Not available	Reference	–
	Yes	Not available	1.28 (0.90–1.82)	–
Had separate kitchen	Yes	–	Reference	Reference
	No	–	1.40 (1.01–1.97)	1.93 (1.14–3.28)
Main cooking fuel	Clean	–	Reference	Reference
	Polluting	–	1.19 (0.72—1.98)	1.98 (1.01–3.92)

*ARR* adjusted risk ratio

Not available for 2006

### Population Attributable Fraction of HAP Factors to Childhood Pneumonia in 2016

In 2016, the PAF calculation showed that 30.9% of childhood pneumonia cases were attributable to not having a separate kitchen and 39.8% to using polluting cooking fuels (Table 4).

**Table 4:** Population attributable fraction (PAF) for adjusted risk factors to childhood pneumonia in 2016

Characteristics	Category	Reference	Proportion of exposure	Relative risk of contracting pneumonia	PAF (%)
Had a separate kitchen room	No	Yes	0.48	1.93	30.86
Cooking fuel	Polluting	Clean	0.75	1.98	39.76

## Discussion

Nepal has reduced under-five mortality by more than 75% since 1990 achieving the target set by MDG (National Planning Commission 2016). The case fatality rate for pneumonia has decreased from 0.4 in 2000/01 to 0.06 in 2013/14 (Ministry of Health et al. 2017). Although the prevalence of childhood pneumonia had reduced in Nepal, the proportion of cases in households using polluting cooking fuels was still high in 2016. There was a shift in the childhood pneumonia risk factors in Nepal between 2006 and 2016, and in 2016 a significant proportion of childhood pneumonia could be attributed to household air pollution. The significantly associated factors such as mother’s occupation and wasted nutritional status in 2006, were no longer significant in 2016. This may be due to the overall decrease in the rate of wasting in children from 7.2% in 2006 to 2.2% in 2016. In 2016, the HAP factors of not having a separate kitchen and using polluting fuels were risk factors for childhood pneumonia. The association of childhood pneumonia in 2016 with the use of polluting cooking fuel and the absence of a separate kitchen indicates that interventions need to focus on installing improved household cooking stoves.

The prevalence of childhood pneumonia reduced to half in households using polluting fuels and declined by more than two-thirds in households using clean fuels between 2006 and 2016. While this trend of decrease appears, the prevalence of childhood pneumonia in households using clean cooking fuels is lower than in households using polluting cooking fuel. The decreasing trend could also be as a result of the enforcement of the Tobacco Control Law in 2010, the improved nutritional status of Nepal’s children between 2006 and 2016, or the introduction of pneumococcal vaccine (Ministry of Health, Nepal 2016). Despite the decrease in number of households not having a separate kitchen and using polluting cooking fuel which could be possibly due to improvements in housing conditions and use of petroleum gases and other newer fuels for cooking food in Nepal, the risk for childhood pneumonia remained high. This clearly calls for further research and intervention to replace the polluting fuels by cleaner fuels in the households.

While the Pneumococcal Congugate Vaccine (PCV) was introduced in early 2015 in west Nepal and July 2015 in the rest of Nepal, we cannot comment on its potential impact on the childhood pneumonia data collected for the NDHS 2016 (Ministry of Health et al. 2017). The coverage of third dose of PCV was less than 50% among children in this survey and was also different based on the sociodemographic characteristics. There are possible differences in housing characteristics of respondents as well. The number of homes with ‘natural’ flooring declined between 2006 and 2011, but rose between 2011 and 2016. This was probably due to the devastating earthquakes of 25 April, 2015 and 12 May 2015 which damaged more than 600,000 homes countrywide beyond repair (Welton-Mitchell et al. 2016). However the effect of the post earthquake situation on rates of childhood pneumonia may not be fully represented as the data collection was done just few months after the earthquake in 2015 for the NDHS 2016 (Ministry of Health et al. 2017).

Prevalence of childhood pneumonia in mountains and hills are higher as the use of solid fuels to heat homes and prevent cold in the winter hill and mountain areas is more common. The use of polluting fuels for cooking varies by social status, geographical location, culture and age (Acharya et al. 2015; Bates et al. 2013). In Nepal, a variety of cooking methods are used by people from different cultures ranging from roasting over an open flame to cooking in an open earthen oven and boiling and steaming (Acharya et al. 2015; Bates et al. 2013). In Nepal, women do most of the household cooking and caring for young children. This means that children are exposed to smoke generated from cooking stoves (Shimada and Matsuoka 2011).

Exposure to HAP is strongly associated with a household’s socioeconomic status (Pant 2012). Cheaper fuel such as charcoal, wood, dung and crop residues produce more smoke and tend to be the ones that socioeconomically poorer people use (Murray et al. 2012). Electric and LPG stoves are the least polluting means of household cooking, but are unaffordable to poorer people. Houses in rural areas are less likely to have smoke vents and chimneys (Acharya et al. 2015). Air-drawing flues may be used to vent out smoke, but these need regular maintenance to prevent malfunctions that can lead to increased HAP (Rhodes et al. 2014).

Interventions that promote cleaner cooking fuels and improved stoves will decrease the impact of HAP on household members (Bruce et al. 2013). The WHO recommends maximum average ambient 24-hour PM_2.5_ exposure of 25 μg/m^3^ (Chafe et al. 2015). Tackling HAP requires a multi-sectoral approach and is a priority of the Sustainable Development Goals (SDGs) (Amegah et al. 2016). Improved cooking stoves (‘smokeless stoves’) are another important intervention to improve the quality of indoor air at household level in selected districts (Singh et al. 2012; Winrock International 2013).

The study has some limitations. The pneumonia prevalence was taken based on the symptoms in the past 2 weeks preceding the surveys, therefore a little caution may be required when discussing prevalence. The symptoms based reporting could have differences in reporting by respondents with different education levels based on their understanding and perception regarding the symptoms. Although we use the term “trends”, we acknowledge that this paper actually compares prevalence from three data points separated by 5 years. The trends would have been different for data collected throughout the year taking all seasons into account such that the prevalence may need to be cautiously interpreted. The NDHS data were cross-sectional and so the incidence of the disease could not be calculated. However, the NDHS has a fairly uniform method of data collection across its surveys. The data collected on childhood pneumonia by the surveys required stratification of data within certain important variables such as wealth. Behavioural risk factors could have been better estimated if a standard definition of HAP had been used during data collection.

## Conclusion

Despite the reduced prevalence of childhood pneumonia between 2006 and 2016, HAP remains a major cause of childhood pneumonia. The study calculated that childhood pneumonia could be reduced by about 31% if kitchens are separated from living area and by 40% if cleaner cooking fuels replace polluting fuels. Achieving this requires interventions in different sectors, especially to promote the use of cleaner fuels. Household behaviour should also be targeted to promote the use of clean energy as one intervention needing a low level of investment.

It is recommended that childhood pneumonia be combatted by promoting (i) the use of separate spaces for cooking to minimize children’s exposure to polluted indoor air; (ii) the use of clean energy for cooking and lighting through social subsidies; and (iii) by improving ventilation in houses that cook with solid fuel.

## Abbreviations

SDG: Sustainable development goal
PAF: Population attributable fraction
HAP: Household air pollution
ICF: Inner city fund
MEASURE DHS: Monitoring and evaluation to assess and use results demographic and health surveys
NDHS: Nepal demographic and health survey
